# CD14 C-159T and early infection with *Pseudomonas aeruginosa *in children with cystic fibrosis

**DOI:** 10.1186/1465-9921-6-63

**Published:** 2005-06-23

**Authors:** AC Martin, IA Laing, G Zhang, S Brennan, K Winfield, PD Sly, SM Stick, J Goldblatt, PN LeSouef

**Affiliations:** 1 School of Paediatrics and Child Health, University of Western Australia, Perth, Western Australia 6001; 2 Division of Clinical Science, Telethon Institute for Child Health Research, Perth, Western Australia 6008; 3 Department of Respiratory Medicine, Princess Margaret Hospital for Children, Perth, Western Australia 6008

**Keywords:** cystic fibrosis, CD14, *Pseudomonas aeruginosa*

## Abstract

Early acquisition of *Pseudomonas aeruginosa *is associated with a poorer prognosis in patients with cystic fibrosis. We investigated whether polymorphisms in CD14, the lipopolysaccharide receptor, increase the risk of early infection. Forty-five children with cystic fibrosis were investigated with annual bronchoalveolar lavage (BAL) and plasma sCD14 levels. Plasma sCD14 levels were significantly lower in children from whom *P.aeruginosa *was subsequently isolated (492.75 μg/ml vs. 1339.43 μg/ml, p = 0.018). Those with the CD14 -159CC genotype had a significantly increased risk of early infection with *P.aeruginosa *suggesting that CD14 C-159T plays a role in determining the risk of early infection with *P.aeruginosa*.

## Introduction

Cystic fibrosis (CF) is the most common serious, monogenic, autosomal recessive disease in Caucasians and results from mutations in the gene encoding the cystic fibrosis transmembrane conductance regulator (CFTR). CF is characterised by variable phenotypic expression, which is not entirely explained by the allelic heterogeneity of the pathogenic CFTR mutations, and there is accumulating evidence that much of this phenotypic diversity is due to the effect of modifier genes [1]. *Pseudomonas aeruginosa*, an environmental organism, is the most important pathogen in patients with CF as chronic infection results in a more rapid decline in lung function and reduced survival [2].

CD14, a key gene of the innate immune system, functions as a receptor for lipopolysaccharide (LPS), a constitutive element of the *P.aeruginosa *cell wall, and is a potential modifier of severity in patients with CF. CD14 is expressed in both a membrane-bound form on macrophages, monocytes and neutrophils (mCD14) and soluble form in serum (sCD14). A polymorphism in the CD14 gene promoter (C-159T) has an allele frequency of approximately 50% in Europeans [3]. The -159C allele is associated with lower circulating levels of sCD14 in healthy children. Higher constitutive levels of mCD14 and sCD14 have been shown to increase the magnitude of airway neutrophil response to LPS [4], whereas CD14 receptor blockade results in a reduction in the deleterious systemic responses that occur in sepsis, due to a reduction in pro-inflammatory cytokines but at the expense of an increased bacterial load [5]. Hence, CD14 may play a pivotal role in determining the balance of infection and inflammation in CF. Increased expression of CD14 may be associated with more inflammation but a reduced bacterial load, whereas reduced expression may result in less inflammation but a greater bacterial load.

We hypothesised that *P.aeruginosa *infection would be acquired earlier in children with the -159CC genotype.

## Methods

A prospective, population-based, cohort of children with CF was recruited from the only tertiary paediatric hospital in Western Australia, which cares for all children with CF in the state. Annual bronchoscopy, performed through a laryngeal mask to minimise the risk of upper airway contamination, was done routinely from diagnosis until 7 years of age in all children with CF. Children diagnosed after the neonatal period, whose first BAL was positive for *P.aeruginosa *were excluded from this analysis, as in this situation it could not be determined when they had initially acquired this pathogen.

All children with a positive BAL culture for *P.aeruginosa*, which was defined as >10,000 CFU/ml, were admitted to hospital for further treatment. Blood was collected for genetic studies and subjects were genotyped for the CD14 promoter polymorphism as previously described [3]. Plasma samples taken at the time of the earliest BAL, when the children were not infected with *P.aeruginosa*, was available for measurement of sCD14 levels in 31 children. Plasma sCD14 levels were determined using a commercially available enzyme-linked immunosorbent assay (ELISA) kit (R&D Systems, Minneapolis, USA).

The Kaplan-Meier survival method was used to explore the difference in the *P.aeruginosa *free survival rates between CD14 genotype groups. As children in the study were not the same age, data was censored for individual children who were known to have not isolated *P.aeruginosa *at the time of their last BAL and this information was incorporated into the analysis. Breslow test (Breslow Generalized Wilcoxon Test) was employed to compare the difference in the survival curves of acquisition of *P. aeruginosa *in the three CD14 genotype groups. Multivariate Cox regression was used to estimate the relative risks after adjustment for potential confounding factors including age, sex, CFTR mutation and nutritional status. Plasma sCD14 levels were analysed by ANOVA and all the statistical analyses were performed using SPSS for Windows (Version 11).

Parents of all participants gave informed consent and the Ethics Committee of King Edward Memorial and Princess Margaret Hospitals, Western Australia, approved the study.

## Results

Forty-five children (22 male), aged 0.6–6.6 years (mean 3.25 years) were studied, of whom 25/45 (55%) were DF508 homozygous and 20/45 DF508 heterozygous. CF was diagnosed at a mean age of 0.23 yrs (95%CI 0.06–0.4 yrs), with 73% diagnosed in the newborn period. There was no significant difference between CFTR mutation (p = 0.74), gender (p = 0.38), nutritional status (weight for height z scores) (p = 0.39) or socio-economic status (p = 0.29) in those who did and did not isolate *P.aeruginosa*. CD14 genotype frequencies at position -159 were: CC 12/45 (27%), CT 20/45 (44%), TT 13/45 (29%). *P.aeruginosa *was isolated from thirteen children (29%) and the mean age of acquisition was 2.26 years (95% CI 1.29–3.25). Subjects with CD14 -159CC appeared to isolate *P.aeruginosa *at a younger age (mean = 1.1 years, 95%CI = 0.2–1.9 years) than -159CT (mean = 2.8 years, 95%CI = 1.3–4.2 years) and TT subjects (mean = 3.3 years), although this difference was not statistically significant (p = 0.19).

**Figure 1:**
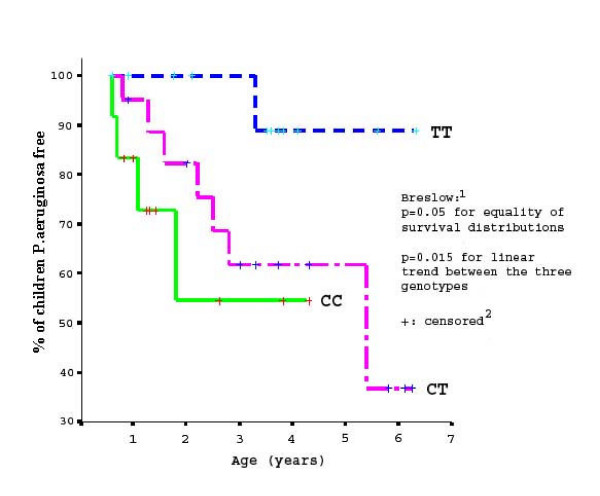
Kaplan-Meier estimates of proportion of children free from *P.aeruginosa *by CD14 C-159T. ^1^Breslow Test (Breslow Generalized Wilcoxon Test) ^2^"Censored" represents the censored observations that arise when the duration of a study is limited.

Figure 1 shows the *P.aeruginosa *free survival curves of the three CD14 genotype groups. The probability of children remaining free of *P.aeruginosa *with -159CC is consistently below that of children who are CT or TT and the curve for children with CT is below that of children with TT. For example, at 2 years of age, the % children remaining uninfected with *P.aeruginosa *is 55% vs. 82% vs. 100% for CC, CT and TT respectively.

Compared with -159TT, children with the CC genotype had a 10-fold (95%CI = 1.09–92.30, p = 0.042) higher relative risk and children with the CT genotype had an intermediate 5.5 fold (95%CI = 0.69–44.63, p = 0.108) higher relative risk of being infected with *P.aeruginosa*. After adjustment for CFTR mutation and nutritional status, the estimated relative risk in children with CC or CT increased further (RR = 13.32, 95%CI = 1.37–129.13, p = 0.025 and RR = 6.0, 95%CI = 0.71–51.03, p = 0.101 respectively). This suggested that independent of CFTR mutation and nutritional status, those with the C allele had a significantly increased risk of being infected with *P.aeruginosa*. In addition, there was a significant linear trend across the three genotype groups, between increasing numbers of C alleles and increasing likelihood of being infected with *P.aeruginosa *(p = 0.015).

Compared to children who subsequently isolated *P.aeruginosa*, those who remained free of *P.aeruginosa *had significantly higher plasma sCD14 levels: 1339.43 μg/ml (95%CI 1096.63–1635.98 μg/ml) vs. 492.75 μg/ml (95%CI 55.7–4359.01 μg/ml), p = 0.018. We found no significant association between plasma sCD14 levels and CD14 C-159T (p = 0.38).

## Discussion

This study showed an association between CD14 -159CC and early acquisition of *P. aeruginosa *in children with CF. Many CF centres promote aggressive treatment regimes to eradicate first isolates of *P.aeruginosa*, and, thus, prospectively identifying a "high risk" group of infants with CF could have substantial clinical benefit.

CD14 receptor activation results in a strong Th1 cytokine response, directed through IL-12, in an attempt to eradicate pathogens. The finding of lower plasma sCD14 levels in children who subsequently isolate *P.aeruginosa *suggests that an inadequate pro-inflammatory response to LPS may place these individuals with CF at greater risk of early *P.aeruginosa *acquisition. The lack of association between CD14 C-159T and plasma sCD14 levels may merely reflect the small number of subjects in this population, but may also indicate that in CF there are factors more critical than CD14 C-159T genotype that determine plasma sCD14 levels.

Although children with higher sCD14 levels may be relatively protected from earlier acquisition of *P.aeruginosa*, when they become colonised they may paradoxically have a worse outcome, as a result of an over-aggressive and ineffectual inflammatory response. These latter patients might potentially benefit from aggressive anti-inflammatory treatment. Thus, the timing of anti-inflammatory therapy would need to be carefully considered, as inflammation might be beneficial in early life by delaying colonisation with *P.aeruginosa*, but harmful once infection is established, due to the over-exuberant inflammatory response. Therapy that modulates the inflammatory response might promote the acquisition of pathogens such as *P. aeruginosa *in young children with different genetic susceptibilities.

The results of this study are based on a limited number of subjects due to the stringent inclusion criteria and the prospective longitudinal design. In addition, the potential role of other single nucleotide polymorphisms in genes involved in innate immunity such as Toll-like receptor 4 Asp299Gly [6], Toll-like receptor 2 Arg753Gln [6] and mannose binding lectin [7], may influence the acquisition of *P.aeruginosa *in children with CF and more detailed investigation of these pathways in CF is indicated. This study identified the potential association between a common genetic variant and the early isolation of *P.aeruginosa*. The CD14 promoter polymorphism may have a central function in determining the age of first isolation of *P.aeruginosa*, with the CC genotype conferring a higher risk of early isolation and the TT genotype being relatively protective, emphasising the importance of understanding the delicate balance between inflammation and infection that exists in CF.
